# A novel DNA methylation marker to identify lymph node metastasis of colorectal cancer

**DOI:** 10.3389/fonc.2022.1000823

**Published:** 2022-10-14

**Authors:** Yingdian Yu, Wenyuan Xue, Zefeng Liu, Shang Chen, Jun Wang, Quanzhou Peng, Linhao Xu, Xin Liu, Chunhui Cui, Jian-Bing Fan

**Affiliations:** ^1^ Department of Biochemistry and Molecular Biology, School of Basic Medical Sciences, Southern Medical University, Guangzhou, China; ^2^ Department of General Surgery, Zhujiang Hosipital, Southern Medical University, Guangzhou, China; ^3^ AnchorDx Medical Co., Ltd., International Bio-Island, Guangzhou, China; ^4^ Department of Pathology, School of Basic Medical Sciences, Southern Medical University, Guangzhou, China

**Keywords:** colorectal cancer, lymph node metastasis, DNA methylation marker, immunohistochemistry, diagnostic method

## Abstract

Lymph node metastasis (LNM) of colorectal cancer (CRC) is an important factor for both prognosis and treatment. Given the deficiencies of conventional tests, we aim to discover novel DNA methylation markers to efficiently identify LNM status of CRC. In this study, genome-wide methylation sequencing was performed in a cohort (n=30) using fresh CRC tissue to discover differentially methylated markers. These markers were subsequently validated with fluorescence quantitative PCR in a cohort (n=221), and the optimal marker was compared to conventional diagnostic methods. Meanwhile, immunohistochemistry was used to verify the effectiveness of the antibody corresponding to this marker in a cohort (n=56). *LBX2* achieved an AUC of 0.87, specificity of 87.3%, sensitivity of 75.7%, and accuracy of 81.9%, which outperformed conventional methods including imaging (CT, PET-CT) with an AUC of 0.52, CA199 with an AUC of 0.58, CEA with an AUC of 0.56. *LBX2* was also superior to clinicopathological indicators including the depth of tumor invasion and lymphatic invasion with an AUC of 0.61and 0.63 respectively. Moreover, the AUC of *LBX2* antibody was 0.84, which was also better than these conventional methods. In conclusion, A novel methylation marker *LBX2* could be used as a simple, cost-effective, and reliable diagnostic method for LNM of CRC.

## Introduction

Colorectal cancer (CRC) is the third most common cancer in the world. Until now, the incidence and mortality rate have increased to the third and the second among all cancers. However, lymph node metastasis (LNM) is the main cause of the increasing mortality in CRC (1). According to National Comprehensive Cancer Network (NCCN) guidelines on the treatment of CRC, surgical operation is still the preferred treatment for CRC, meanwhile, lymph node dissection is recommended whenever there is an opportunity to remove the tumor (2). Although lymph node dissection could reduce the recurrence of CRC, the patients without LNM could not benefit from lymph node dissection, but it could bring many complications such as postoperative intestinal adhesion, intestinal obstruction, lymphatic leakage, sexual dysfunction, and postoperative bleeding, which lead to excessive medical treatment (3).

Currently, the clinical diagnosis of LNM of CRC mainly relies on imaging including computed tomography (CT) and positron emission tomography-computed tomography (PET-CT), or clinicopathological characteristics including depth of tumor invasion, ulceration, lymphatic vascular invasion, etc. (4, 5). In addition, some clinical serological indicators such as carcinoembryonic antigen (CEA) and carbohydrate antigen 199 (CA199) could also be used as a basis for LNM of CRC (6). However, the accuracy and reliability of these methods is not ideal. This may be the primary reason why the NCCN guidelines on surgical treatment of CRC recommend lymph node dissection, despite its potential for postoperative complications.

DNA methylation is one of the important epigenetic modifications. It has been proved that abnormal DNA methylation is related to cancer. During tumorigenesis, changes in DNA methylation patterns may be easily detected, thus tumor-related methylation markers have more accurate and direct effects on cancer diagnosis (7). So far, many studies on DNA methylation of CRC are based on early diagnosis and prognosis. In terms of early diagnosis, methylation of the promoter of *RASSF1A* (8), methylation of the CpG of *Caveolin-1 (* 9), hypomethylation of transcription suppressor *HES1 (* 10), hypomethylation of histone lysine methyltransferase encoding gene *SMYD3 (* 11) are proved to be associated with CRC. In terms of prognosis assessment, methylation of the promoter of *CDX2* is an independent indicator of prognosis of CRC (12), and methylation of the promoter of RAI2 is a poor indicator of prognosis of CRC (13).

Our previous work has demonstrated that the methylation markers of *KCNJ12*, *VAV3-AS1*, and *EVC* could be used as the basis for stage and stratification of CRC, with an area under curve (AUC) of 0.87, sensitivity of 83.0%, and specificity of 71.2% (14). Currently, it is common to take CRC tissue under colonoscopy for preoperative diagnosis in clinical practice. By obtaining CRC tissue samples, this study aims to identify LNM status of CRC by discovering novel DNA methylation markers, which could be used for the formulation of clinical treatment plans and prognosis evaluation of CRC.

## Methods

### Study design and patient recruitment

**Figure 1:**
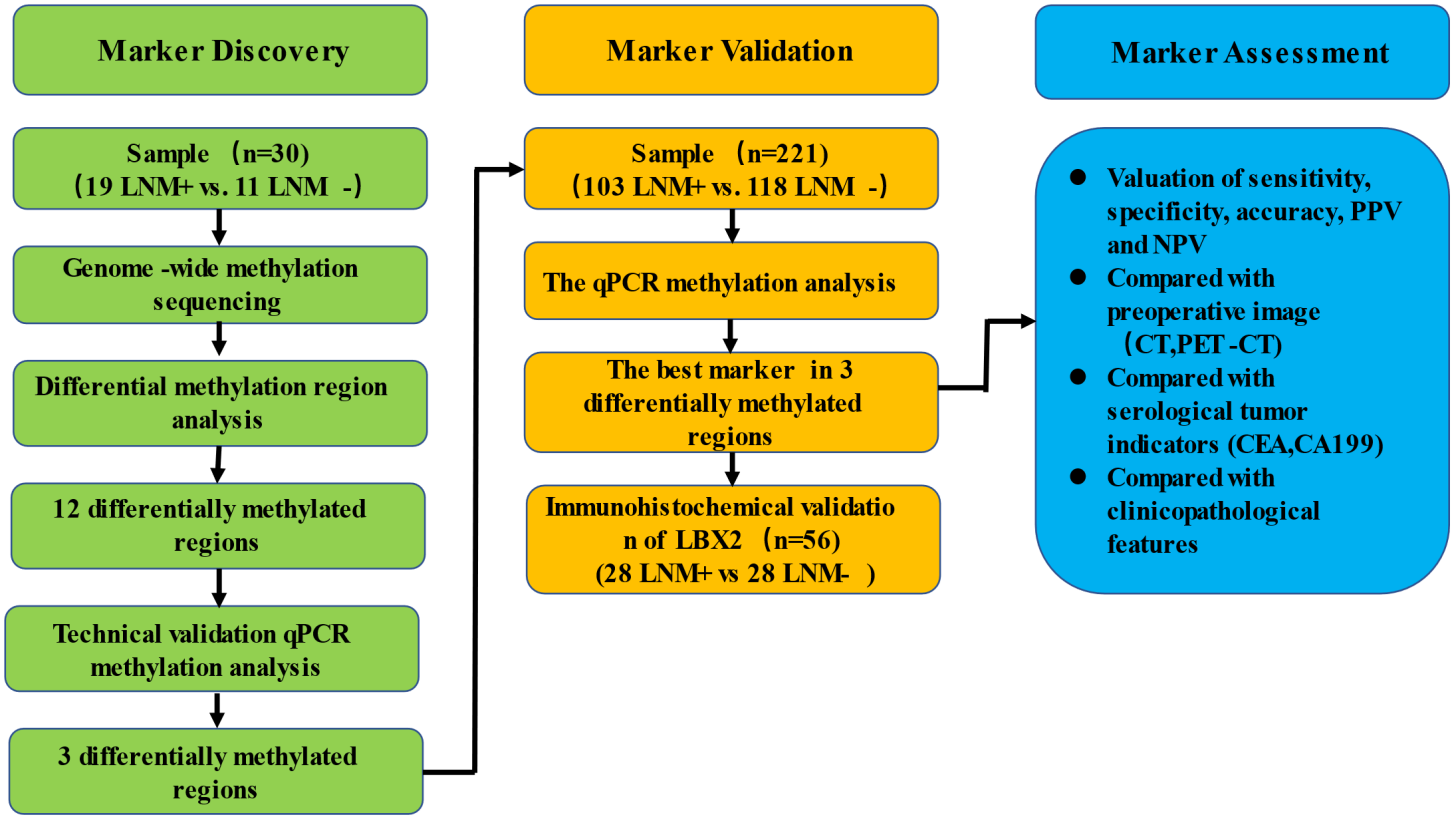
Schematic workflow of the study design.

In this study, a three-phase strategy was designed (**Figure 1**), which included a marker discovery cohort (n = 30, fresh frozen (FF) tissue samples) and a marker validation cohort (n =221, FF and formalin-fixed paraffin-embedded (FFPE) tissue samples). The proportion of tumor in all tissue samples was more than 60%, which was obtained by two qualified pathologists on observing paraffin sections with high-power microscopy. Genome-wide methylation sequencing was performed on 30 FF tissue samples from the marker discovery cohort to identify LNM-specific methylation markers. These methylation markers were verified by fluorescence real-time quantitative PCR (qPCR) from the marker validation cohort (n=221). The optimal methylation markers were selected to compare with imaging (CT and PET-CT), serological indicators (CEA and CA199) and clinicopathological characteristics in a validation cohort. All CRC patients were recruited from Zhujiang Hospital, Southern Medical University. CRC samples (FF, n=76; FFPE, n=182) were derived from January 2017 to March 2020. Samples with less than twelve lymph nodes (15) and failed DNA quality control (n=37) were excluded from the study. Tissue sample of CRC patients was tumor surgical specimens before radiotherapy or chemotherapy. And these samples corresponding to pathological reports and LNM status were confirmed by at least two gastrointestinal pathologists. The clinicopathological characteristics containing age, gender, depth of invasion (t-stage of TMN), tumor size, lymphatic vessel invasion (LVI), blood vessel invasion (BVI), neural invasion (NI) and ulceration were shown in **Table 1**.

**Table 1:** Characteristics of CRC patients in the validation cohorts.

Characteristics		LNM-	LNM+	P value
		n = 118 (%)	n = 103 (%)	
Age				0.700
	<55	25 (21.2)	26 (25.2)	
	≥55	93 (78.8)	77 (74.8)	
Gender				0.910
Depth of invasion (t-stage)	Male	69 (58.5)	42 (40.8)	0.003
	Female	49 (41.5)	61 (59.2)	
	T1 T2 T3 T4	4 (3.40) 19 (16.1) 57 (48.3) 38 (32.2)	1 (1.00) 8 (7.80) 43 (41.7) 51 (49.5)	
Tumor size (cm)				0.203
	<5	59 (50.0)	62 (60.2)	
	≥5	59 (50.0)	41 (39.8)	
Lymphatic vessel invasion				<0.001
	Yes	14 (11.9)	39 (37.9)	
	No	104 (88.1)	64 (62.1)	
Blood vessel invasion				<0.001
	Yes	17 (14.4)	38 (36.9)	
	No	101 (85.6)	65 (63.1)	
Neural invasion				0.003
	Yes	34 (28.8)	50 (48.5)	
	No	84 (71.2)	53 (51.5)	
Ulceration				0.361
	Yes	65 (55.1)	63 (61.2)	
	No	53 (44.9)	40 (39.8)	

LNM+, samples of CRC patients with lymph node metastasis; LNM−, samples of CRC patients without lymph node metastasis.

### Discovery of methylation markers

30 FF tissue samples of CRC (19 LNM+, 11 LNM-)were collected to identify differential methylation markers. Next, we independently constructed a genome-wide methylation library using TruSeq^®^ Methyl Capture EPIC Library Prep Kit (Illumina, USA, Catalog No. Fc-151-1002). After EPIC library was quality-assured with Agilent High-Sensitivity DNA Kit (Agilent, USA, Catalog No. 5067-4626), high-throughput sequencing was performed on Illumina X-TEN platform.

### DNA extraction, bisulfite treatment

Genomic DNA was extracted from FF tissue samples and FFPE tissue samples with AllPrep DNA/RNA Mini Kit (Qiagen, Germany, Catalog No. 80204) and AllPrep DNA/RNA FFPE Kit (Qiagen, Germany, Catalog No. 80234). Subsequently, the extracted DNA was quantified by the qubit dsDNA Customs Assay Facility (Thermal Fisher Science, USA, Catalog No.Q32851). The quality controlled criteria of CRC samples required that DNA content was more than 100 nanograms and the main band of agarose gel electrophoresis exceeded 500 bps. 50 nanograms of genomic DNA was taken from each sample, and EZ-96-DNA Methylation Direct MagPrep Kit (Zymo Research, USA, Catalog No. D5044) was used for bisulfite treatment of DNA.

### Methylation analysis by fluorescence qPCR

The primer and probe sequences of the selected genes were designed through the biological software Beacon Designer V8.14. Fluorescence qPCR was used for methylation analysis in a validation cohort (n=221,103 LNM+ and 118 LNM-) (16). qPCR methylation analysis was performed on the Quant Studio 3 Real-Time PCR System (Thermo Fisher, USA). Based on our previous study (14), *ACTB* was selected as an internal reference gene. △Ct value obtained by fluorescence qPCR was used to indicate the methylation level of the target gene (△Ct= Ct value of the target gene - Ct value of the reference gene). If the Ct value is not present, the Ct value was set to 40.

### Immunohistochemical analysis

Immunohistochemistry (IHC) was subsequently performed on the optimal genes validated by fluorescence qPCR. A total of 56 CRC paraffin sections (28 LNM+, 28 LNM-) were colllected for immunohistochemical analysis. First, the 2μm thick paraffin sections were roasted at 65°C for 1 hour, dewaxed with xylene and 100% ethanol, repaired with citrate buffer solution (PH 6.0) for 3 minutes under high pressure, and incubated with 3% H202 for 10 minutes. Next, the paraffin sections were sealed with goat serum for 30 minutes, and the primary antibody *LBX2* (Bioss, Beijing, China) was diluted at 1:100 and incubated in a metal bath at 37°C for 1 hour. After washing with phosphate buffered saline PBS (PH 7.6), enzyme-labeled sheep anti-mouse/rabbit IgG polymer (Second Antibody, GeneTech, Shanghai, China) was selected to incubate at 37°C for 30 minutes. Peroxidase activity was cultured with 3, 3-diaminobenzidine hydrochloride (DAB) in sterile H2O2 solution for 2 minutes. Finally, nuclear re-staining was performed with Mayer hematoxylin solution. All the slices were independently examined by two observers. The positive composite score was used in this study, which was the staining intensity multiply the percentage of positive cells. The staining intensity is classified into four levels. No staining was rated 0 point, light yellow was rated 1 point, pale brown was rated 2 points, brown was rated 3 points (**Figures 3A-D**). In addition, the percentage of positive cells was evaluated as 0 points for 0 ~ 5%, 1 point for 6% ~ 25%, 2 points for 26% ~ 50%, 3 points for 51% ~ 75% and 4 points for >75%.

### Comparison of DNA methylation markers and clinicopathological features, imaging, serological indicators

Eight clinicopathological variables were included in univariate analysis to explore their correlation with LNM. Variables with P value less than 0.05 were included in a multivariate analysis. Stepwise regression was used to assess 95% confidence interval (CI) of odds ratio (OR) values to identify independent predictors. DNA methylation marker was compared to the selected clinicopathological indicators, imaging and serological indicators (CEA and CA199) by the area under the receiver operating characteristic (ROC) curve including the specificity, sensitivity, accuracy, positive predictive value (PPV), and negative predictive value (NPV).

### Statistical analysis

R package DSS(2.0.16) of ComplexHeatmap and Corrplot were used for unsupervised hierarchical clustering and correlation analysis, pROC (1.16.1) was used for ROC, AUC and AUC confidence interval calculations, ggplot2 (3.2.1) and RColorBrewer (1.1.2) were used for visualization of figures. Differences between 2 groups were analyzed with the unpaired Student’s t test (2-tailed tests), and 1-way ANOVA followed by Dunnett’s multiple comparisons tests when more than 2 groups were compared. Pearson’s χ2 test was used to analyze the clinical variables on sensitivity and specificity. Univariate and multivariate logistic regressions were used to evaluate the clinicopathological variables. Comparison of AUC values were conducted by Hanley and McNeil tests or DeLong test, when appropriate. The AUC values, sensitivity, specificity, and accuracy of methylation maker *LBX2*, clinicopathological features, serologic tumor markers and image in detecting LNM of CRC were used for comparison. A p value < 0.05 on two sides of all hypothesis tests were considered statistically significant.

## Results

### Genome−wide screening of DNA methylation markers to detect LNM in CRC tissue samples

**Figure 2:**
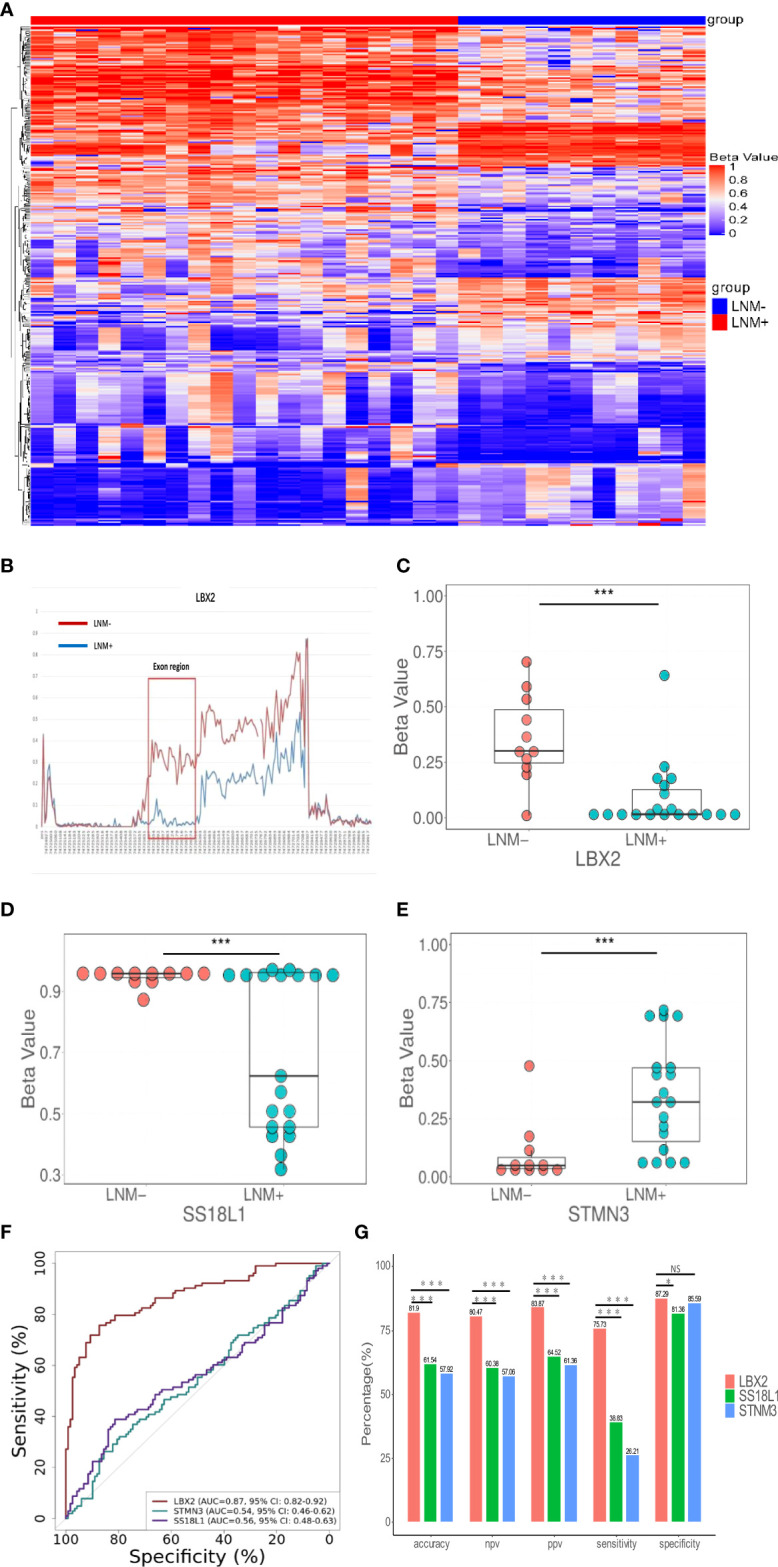
The discovery of DNA methylation markers to detect LNM in CRC tissue. **(A)** In the discovery cohort, an unsupervised hierarchical cluster analysis was based on differential methylation between LNM+(n=19) and LNM-(n=11), with each column representing a patient and each row representing a CpG marker. **(B)** Methylation levels of exon region of *LBX2* in LNM- and LNM+ groups in CRC **(C-E)**. The methylation level distribution of *LBX2, STMN3*, and *SS18L1* between LNM+(n=19) and LNM-(n=11) was represented by the β value from genome-wide methylation sequencing in the discovery cohort. **(F)** ROC curve of three methylation markers. The effectiveness of three DNA methylation methods was evaluated by comparing AUC values. **(G)** The accuracy, NPV, PPV, sensitivity and specificity of three methylation marker were compared respectively. *p < 0.05, **p < 0.01 and ***p < 0.001. NS, not statistically significant.

A schematic workflow of the study design is shown in **Figure 1**. To identify LNM-specific DNA methylation markers in CRC, we first performed genome-wide methylation analysis containing 3.34 million CpG sites on fresh tissue samples from LNM+ group (n=19) and LNM- group (n=11). A total of 734 CpG sites with differential methylation were found (p<0.001, β value difference ≥0.15). The unsupervised heretical clustering showed that LNM+ and LNM- were clearly distinguished by majority of specific DNA methylation markers (**Figure 2A**). Based on these methylation sites, we further analyzed the differential methylation region (DMR) status and screened out twelve markers with DMR status. There were three hypomethylation markers including *LBX2, SS18L1, CYTH2* in LNM + group, meanwhile, there were nine hypermethylation markers including *ACHE、RPS15、APC2、BAHCC1、LEFTY1、RTN4RL2、KCNQ1、STMN3、LINC01072* in LNM+ group.

Our primary goal was to develop a simple methylation-specific qPCR to detect LNM status (16). These twelve markers were further verified by qPCR in a cohort (n=65) (**Supplemental material: Figure S2**). However, nine markers were excluded due to low AUC value and inconsistent methylated patterns. Only three markers including *LBX2, STMN3, SS18L1* showed higher AUC values and consistent methylated patterns in sequencing and methylation-specific qPCR analysis, and significantly differentiated LNM+ from LNM- in the same samples (**Figures 2C–E**). In addition, we found exons of *LBX2* included significant methylation differences between LNM+ and LNM-(**Figure 2B**). To sum up, these results indicated that these three methylation markers and qPCR-based analysis were reliable and could be used for large-scale cohort analysis.

### *LBX2* had the best test performance in three target methylation makers

Through the biological software Beacon Designer V8.14, the information about three target genes (*LBX2, STMN3, SS18L1*) was input to set appropriate conditions. The primer and probe sequence were shown in **Table 2**. Based on validation of 221 tissue samples, *LBX2* achieved an AUC of 0.87 (95%CI 0.82-0.92, p<0.001), specificity of 87.3%, sensitivity of 75.7%, accuracy of 81.9%. *STMN3* achieved an AUC of 0.54 (95%CI 0.46-0.61, p=0.30), specificity of 85.6%, sensitivity of 26.2%, and accuracy of 57.9%. *SS18L1* achieved an AUC of 0.56 (95%CI 0.48-0.63, p=0.15), specificity of 81.4%, sensitivity of 38.8%, and accuracy of 61.5%. The comparison of these three methylation markers was shown in **Figure 2F, G**. Obviously, *LBX2* had higher efficiency in accuracy, specificity, sensitivity and AUC compared to other two methylation markers.

**Table 2:** Designed primer and probe sequences of target genes.

Gene	Forward primer 5’→3’	Reverse primer 5’→3’	Probe 5’→3’
*LBX2*	CGTTTAGTGTTGCGTTAAGGGTTT	AAAATCGAATCTTTCCGAATAACCAAA	TCCGCTCCAAACCACTCTCTTCTCGAAA
*STMN3*	TATCGTTTTGGGTTTATTACGGTTATCG	AACGTAAAACGCGATCCCTCG	ACAAACACCAAACCGAACGCGACTAAATCC
*SS18L1*	GGTTTTGAGCGTCGTTTATATGTTTT	CGAACAACATAACGCATCTATATATAAAAC	AAACCACGACACACCCTCTACTTCCTCAAA

### *LBX2* antibody could identify LNM status by IHC

**Figure 3:**
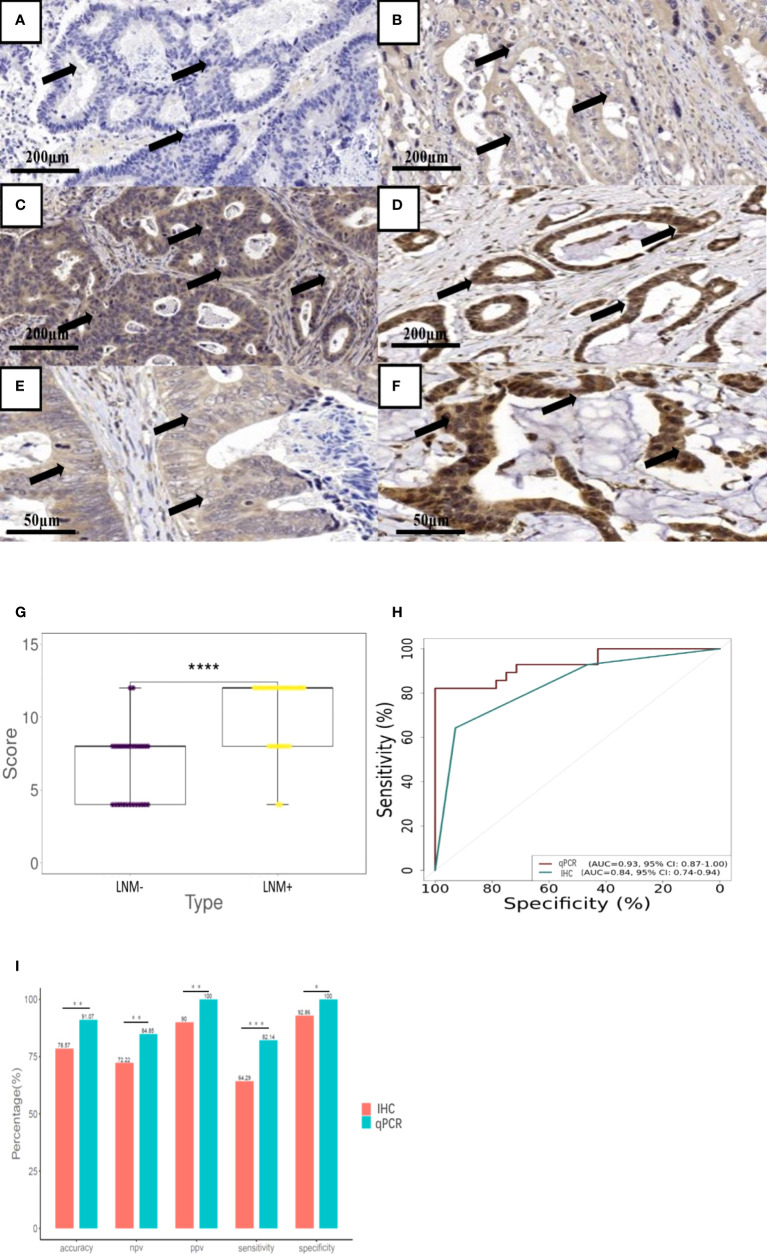
The performance of antibody *LBX2* to detect LNM in CRC tissue. **(A–D)** Immunohistochemical staining depth corresponding to the score. Stained samples (1:100 diluted concentration & 100X Magnification) were divided into four grades. (**A** 0=non-staining **B** 1=light yellow, **C** 2=pale brown **D** 3=brown). **(E)** Faint yellow stained samples (1:100 diluted concentration & 400X Magnification) was considered as LNM- CRC. **(F)** Brown staining samples (1:100 diluted concentration & 400X Magnification) was considered as LNM+ CRC. **(G)** The level of *LBX2* antibody was compared between LNM+(n=28) and LNM-(n=28). **(H)** ROC curve of two methods (IHC&. qPCR). The effectiveness of these two methods was evaluated by comparing AUC values. **(I)** The accuracy, NPV, PPV, sensitivity and specificity of two methods (IHC&. qPCR) were compared respectively. *p < 0.05, **p < 0.01, ***p < 0.001 and ****p<0.0001.

IHC of *LBX2* antibody was performed on CRC tissue section. These sections were amplified by 400 times, the staining of cancer cells was mainly observed. LNM- tissue sections (**Figure 3E**) showed light staining of cancer cells. However, LNM+ tissue section (**Figure 3F**) showed deep staining of cancer cells, especially in the nucleus where there was dark brown, presenting strong positive. In addition, the immunohistochemical score of LNM+ group was significantly higher than that of LNM- group (p<0.001) (**Figure 3G**). Comparing IHC with qPCR in a cohort (LNM+, n=28 and LNM-,n=28), the AUC of IHC was 0.84 (95%CI 0.74-0.94, p<0.001), but qPCR achieved an AUC of 0.93 (95%CI 0.89-0.97, p< 0.001) (**Figure 3H**). Moreover, the specificity, sensitivity and accuracy of the two methods were compared. The specificity, sensitivity and accuracy of immunohistochemical method were 92.9%, 64.3% and 78.6%, respectively. However, the qPCR method achieved a specificity of 100%, sensitivity of 82.1% and accuracy of 91.1% (**Figure 3I**). Obviously, both methods had good discrimination efficiency, despite the qPCR method was better than IHC.

### *LBX2* showed stable table discriminative efficacy in different classification

**Figure 4:**
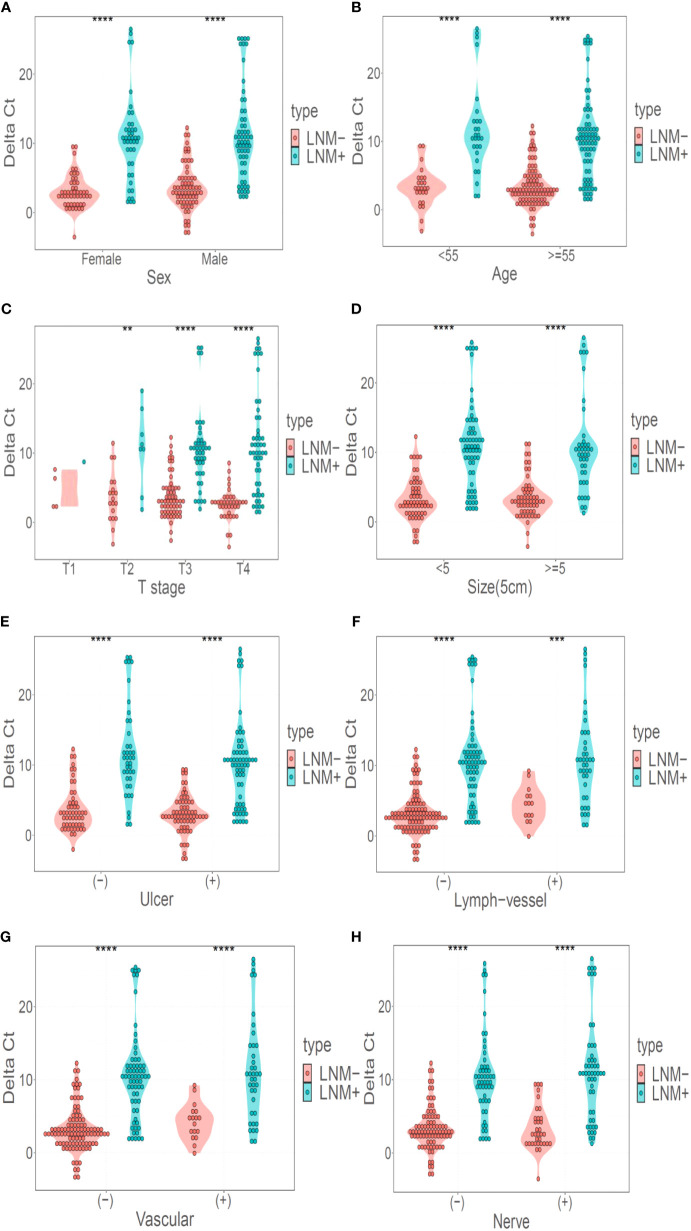
*LBX2* has a good discriminative effect in different classification. **(A)** Performance of *LBX2* in the male and female groups. **(B)** Performance of *LBX2* in age less than 55 years and age more than 55 years groups. **(C)** Performance of *LBX2* in t_1_, t_2_, t_3_, t_4_-staging. **(D)** Performance of *LBX2* in tumor size less than 5cm and tumor size more than 5cm groups. **(E)** Performance of *LBX2* in ulcerative and non- ulcerative groups. **(F)** Performance of *LBX2* in lymph-vessel invasion and non-lymph-vessel invasion groups. **(G)** Performance of *LBX2* in vascular invasion and non- vascular invasion groups. **(H)** Performance of *LBX2* in nerve invasion and non-nerve invasion. *p < 0.05, **p < 0.01, ***p < 0.001 and ****p < 0.0001.

DNA methylation marker *LBX2* could identify LNM of CRC well in both male and female populations (P < 0.001) (**Figure 4A**). Similarly, *LBX2* had good discrimination effect in the group under 55 years old or the group over 55 years old (P < 0.001) (**Figure 4B**). *LBX2* could identify LNM of CRC well at T3 and T4 stages (P < 0.001) and T2 stage (P < 0.05). However, the strength of evidence was weak due to the small sample size at T1 stage (**Figure 4C**). *LBX2* also could identify LNM of CRC in tumor diameter less than 5cm and tumor diameter more than 5cm (P < 0.001) (**Figure 4D**). In different clinicopathological groups, *LBX2* could identify LNM of CRC well in both ulcerated and non-ulcerated groups, both lymphatic and non- lymphatic invasion groups, both vascular and non-vascular invasion groups, and both neural and non-neural invasion groups (P < 0.001) (**Figures 4E-H**). In conclusion, DNA methylation marker *LBX2* had stable performance in different groups of each factor.

### *LBX2* was superior to clinicopathological features in distinguishing LNM

**Figure 5:**
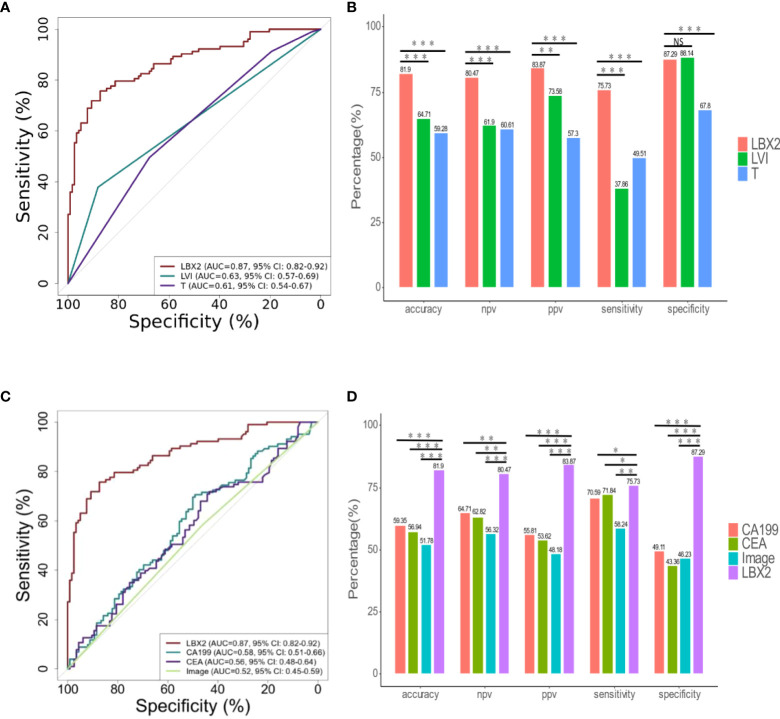
The comparison of *LBX2* and traditional indicators (clinical pathology, CA199, CEA, and image) to detect LNM of CRC. **(A)** ROC curve of three indicators (*LBX2*, LVI, and T). The effectiveness of three indicators was evaluated by comparing AUC values. **(B)** The accuracy, NPV, PPV, sensitivity and specificity of these three indicators were compared respectively. **(C)** ROC curve of four indicators (*LBX2*, CA199, CEA and image). The effectiveness of four indicators was evaluated by comparing AUC values. **(D)** The accuracy, NPV, PPV, sensitivity and specificity of these four indicators were compared respectively. *p < 0.05, **p < 0.01 and ***p < 0.001. NS: not statistically significant.

The relation between clinicopathological features and LNM status was further analyzed. Clinicopathological features included gender, age, depth of tumor invasion (t-stage of TMN), tumor size (demarcated by 5cm), ulcerative, LVI, BVI, and NI. In univariate analysis, there were four factors associated with LNM, including t-stage (OR 1.78, 95% CI 1.23-2.63, p<0.01), LVI (OR 4.52,95%CI 2.33-9.63,p<0.001), BVI (OR 3.47,95%CI 1.84-6.80, p<0.001), NI (OR 2.33,95%CI 1.34-4.09, p<0.01). (**Table 3**); Taking these four factors into account in multifactorial analysis, only LVI (OR 6.41,95%CI 1.31-47.66, p<0.05) and t-stage (OR 1.71,95%CI 1.15-2.61, p<0.01) were related to LNM. Therefore, among these eight clinicopathological features, LVI and t-stage were closely associated with LNM. Next, compared to LVI and t-stage, the *LBX2* achieved an AUC of 0.87 (95%CI 0.82-0.92, p<0.001), accuracy of 81.9%, sensitivity of 75.7%, and specificity of 87.3%. Turning to other two clinicopathological features, the LVI achieved an AUC of 0.63 (95%CI 0.57-0.69, p<0.001), accuracy of 64.7%, sensitivity of 37.9%, and specificity of 88.1%. while the t-stage achieved an AUC of 0.61(95%CI 0.54-0.67, p<0.01), accuracy of 59.3%, sensitivity of 49.55%, and specificity of 67.8% were 59.3%, 49.5%, and 67.8%. Compared to *LBX2*, *LBX2* was clearly superior to clinicopathological features (**Figures 5A, B**).

**Table 3:** Univariate and multivariate logistic regression analyses associated with LNM.

Characteristics	Univariate analysis		Multivariate analysis	
	OR (95%CI)	P value	OR (95%CI)	P value
Gender (Male vs. Female)	1.03 (0.60-1.76)	0.91		
Age (≤ 55 vs. > 55)	1.00 (0.97-1.02)	0.70		
t-stage (1,2 vs.3,4)	1.78 (1.23-2.63)	<0.01	1.71 (1.15-2.61)	<0.01
Tumor size cm (> 5 vs. ≤ 5)	1.00 (0.99-1.00)	0.20		
Ulceration (Presence vs. Absence)	1.28 (0.75-2.20)	0.36		
LVI (Presence vs. Absence)	4.52 (2.33-9.24)	<0.001	6.41 (1.31-47.66)	<0.05
BVI (Presence vs. Absence)	3.47 (1.84-6.80)	<0.001	0.57 (0.08-2.71)	0.51
NI (Presence vs. Absence)	2.33 (1.34-4.09)	<0.01	1.52 (0.82-2.81)	0.18

OR, odds ratio; CI, confidence interval.

### *LBX2* was superior to CEA, CA199, imaging in distinguishing LNM

The relation between CA199, CEA, imaging and LNM status was analyzed. The AUC of CA199 was 0.58 (95%CI 0.51-0.66), with the specificity of 49.1%, the sensitivity of 70.6%, and the accuracy of 59.3%. Moreover, the AUC of CEA was 0.56 (95%CI 0.48-0.64), with the specificity of 43.4%, the sensitivity of 71.8% and the accuracy of 56.9%. In addition, the AUC of imaging (CT and PET-CT) was 0.52 (95%CI 0.45-0.59), with the specificity of 46.2%, the sensitivity of 58.2%, and the accuracy of 51.8%. Compared to these three conventional methods, the AUC of *LBX2* was 0.87 (95%CI 0.82-0.92, p<0.001),with the specificity of 87.3%, the sensitivity of 75.7%, and the accuracy of 81.9%, which was better than these current clinical examination (**Figures 5C, D**).

## Discussion

DNA methylation profiles may represent relatively stable long-term programming of the genome and underlying cellular functions, which is a reliable method of the diagnosis of cancer occurrence and progression (17). Therefore, in this study, genome-wide methylation sequencing on CRC tissues (n=30) was performed and three LNM related specific methylation markers were selected. These three methylation markers were further validated by a large retrospective cohort of 221 tissue samples. We found that a qPCR-based methylated marker *LBX2* had the best discriminative performance for the diagnosis of LNM, which was superior to traditional clinicopathological features, as well as imaging, CEA, and CA199. *LBX2* also had stable discriminative efficacy in different groups including age, sex, tumor size, depth of tumor invasion and clinicopathological feature. At the same time, the antibody corresponding to *LBX2* also showed good performance in differentiating LNM of CRC in immunohistochemical validation. In addition, a more comprehensive approach was used to analyze CRC-associated LNM differential methylation sites, which covered more than 3.34 million CpG sites, accounting for 97.3% of the CpG islands in the genome. To date, few studies have used such a wide range of genome-wide methylation strategies to discover methylation markers for the diagnosis of LNM in CRC.

In previous similar studies, Tsuyoshi Ozawa et al. used microRNA sequencing data from the Cancer Genome Atlas (TCGA) and analyzed a five microRNAs model (*MIR32, MIR181B, MIR193B, MIR195, and MIR411*) that could distinguish LNM in t1-t2 CRC. This verified model achieved an AUC of 0.74 (18), lower than that of the single DNA methylation marker *LBX2* (AUC 0.87) in this study. Moreover, Ailin Qu et al. found a four microRNAs model (*Mir-122-5p, Mir-146B-5p, Mir-186-5p and Mir-193a-5p*) related to LNM status of CRC from high-throughput sequencing data of CRC tissues (n=20), and it showed that the AUC of the four microRNAs model was 0.88 through a verification cohort (n=198) (19), which was similar to the detection efficiency of DNA methylation sites in our experiment. However, compared with RNA markers, DNA methylation markers are more stable as diagnostic biomarkers and relatively stable clinical specimens, which are not easily degraded. Therefore, it is easier to be applied to clinical practice.

Interestingly, *LBX2* could be used to identify the LNM status of CRC not only in qPCR verification, but also in immunohistochemical verification, which indicated that *LBX2* played a significant role in the differentiation of both molecular level and protein level. *LBX2* is located on chromosome 2, which starts at 74725882 and terminates at 74726332, with a total length of 451bp and containing 34 CG. In the LNM- and LNM+ groups, there were significant differences in the methylated levels of *LBX2* in exon region (**Figure 2B**). Due to the low methylated level of *LBX2* in the LNM+ group, it would be overexpressed in the process of protein translation. On the contrary, *LBX2* in the LNM- group has a high methylated level, which results in low expression in the process of protein translation. This view is well explained by our immunohistochemical verification. Subsequently, compared qPCR with IHC in 56 CRC samples, it is clearly that qPCR had better performance (AUC 0.93 vs. 0.84). Apparently, qPCR method had better differentiated efficiency because of more sophisticated level quantification from the qPCR instrument, while IHC was manually assessed and divided into only four grades according to the depth of staining. Because IHC examination is cheaper and easier to generalize, both methods could be applied flexibly for clinical practice.

Currently, there are few studies on gene *LBX2* related to CRC. Some researchers have found that *LBX2-AS1* could promote cell proliferation, migration and invasion through *Mir-4766-5P* mediated *CXCL5* upregulation in gastric cancer (20). In addition, it has been reported that knockout of *LBX2-AS1* in hepatoma cells could reduce its proliferation (21). Moreover, it has been proved that Zinc-finger E-box binding homeobox 1 (*ZEB1*) could induce upregulation of *LBX2-AS1* to enhance the stability of *ZEB1* and *ZEB2*, which could promote the migration and mesenchymal transformation of esophageal squamous cell carcinoma (22). The potential biological pathways of *LBX2* upregulation remain to be proved further. It is widely accepted that *LBX2* may be involved in the positive regulation of Wnt signaling pathway, which is active in the nucleus. Meanwhile, Wnt signaling pathway may be a complex protein action network, whose function not only participates normal physiological processes and embryonic development, but also induces cancer (23). Wnt signaling pathway mainly occurs in intestinal epithelial cells. Under normal conditions, colonic epithelial cells could bind secretory Frizzled related proteins (SFRP) to inhibit Wnt signaling. Once SFRP is silenced under epigenetic regulation, the Wnt signaling pathway would be activated and other molecules in the signaling pathway may mutate, which promotes cell proliferation and inactivation of cells into differentiation and results in the occurrence and invasion of tumors (24).

Although there are a few clinical methods to identify LNM of CRC, the discrimination efficiency of these methods is generally limited. In this study, it had been found that the AUC of imaging, CEA, and CA199 were only 0.52, 0.56 and 0.58, respectively. In addition, the AUC of LVI and the depth of tumor invasion in clinicopathology was 0.63 and 0.61. This may be the main reason that the CRC surgical treatment guidelines suggest we should remove intact tumor with lymph node dissection (2). In fact, the incidence of LNM in many CRC patients, especially those in t1-t2 stage, is only 16% (18). Therefore, excessive medical treatment frequently exists on many CRC patients. Since *LBX2* achieved an AUC of 0.87, which is significantly superior to the current clinical diagnostic methods, meanwhile, DNA samples are more stable than RNA samples. Therefore, DNA methylation marker *LBX2* is easy to be transformed into clinical application and it has the opportunity to become a novel clinical indicator for the identification of LMN of CRC.

Turning to clinical application of *LBX2* in the future, the LNM status of CRC could be determined by immunohistochemical analysis or qPCR analysis of biopsy tissue obtained by colonoscopy. In addition, because CRC tumor cells are easily shed into stool and blood, we could also extract DNA of stool and ctDNA of blood and detect LNM of CRC by *LBX2* probe. This makes it possible to identify LNM of CRC early by minimally invasive or noninvasive methods.

## Conclusion

In conclusion, a novel DNA methylation marker *LBX2* could be used as a simple, cost-effective, easy-to-implement, and reliable diagnostic method for LNM of CRC compared to traditional methods, it holds the potential to provide a better clinical diagnosis for the precise treatment of CRC.

## Data availability statement

The datasets presented in this study can be found in online repositories. The names of the repository/repositories and accession number(s) can be found in the article/**Supplementary Material**.

## Ethics statement

The studies involving human participants were reviewed and approved by Ethics Committee of Zhujiang Hospital, Southern Medical University; Southern Medical University. The patients/participants provided their written informed consent to participate in this study. Written informed consent was obtained from the individual(s) for the publication of any potentially identifiable images or data included in this article.

## Author contributions

J-BF and CC conceived, designed, and directed the study. YY designed the experiments and developed the methodology. YY and WX completed experimental work. YY, JW, LX, and XL performed the analyses and interpretation of data. YY, ZL, SC, and QP acquired the patient samples and information. YY and J-BF wrote and critically reviewed the manuscript. All authors reviewed and approved the final manuscript. All authors contributed to the article and approved the submitted version.

## Funding

This study was supported by Science and Technology Planning Project of Guangdong Province, China(Grant NO.2017B020226005), the 2020 Guangzhou Development Zone International Science and Technology Cooperation Project(NO:2020GH15), Scheme of Guangzhou Economic and Technological Development District for Leading Talents in Innovation and Entrepreneurship (Grant NO.2017-L152), Scheme of Guangzhou for Leading Talents in Innovation and Entrepreneurship(Grant NO.2016007), and Scheme of Guangzhou for Leading Team in Innovation(Grant NO.201909010010).

## Acknowledgments

I thank the staff of the J-BF’s Laboratory in Southern Medical University and the AnchorDx R&D team (AnchorDx Medical Co., Ltd) for their excellent technical assistance. I also thank CC (Department of General Surgery, Zhujiang Hospital, Southern Medical University, Guangzhou, China) for providing valuable clinical samples.

## Conflict of interests

The authors J-BF, JW, XL, LX are current employees of AnchorDx Medical Co., Ltd or AnchorDx, Inc.

The remaining authors declare that the research was conducted in the absence of any commercial or financial relationships that could be construed as a potential conflict of interest.

## Publisher’s note

All claims expressed in this article are solely those of the authors and do not necessarily represent those of their affiliated organizations, or those of the publisher, the editors and the reviewers. Any product that may be evaluated in this article, or claim that may be made by its manufacturer, is not guaranteed or endorsed by the publisher.

## Abbreviations

LNM, Lymph node metastasis; CRC, colorectal cancer; IHC, Immunohistochemistry; NCCN, National Comprehensive Cancer Network; CT, Computed tomography; PET-CT, Positron emission tomography-computed tomography; CEA, Carcinoembryonic antigen; CA199, Carbohydrate antigen 199; FF, Fresh frozen; FFPE, Paraffin-embedded; qPCR, Quantitative PCR; LVI, Lymphatic vessel invasion; BVI, Blood vessel invasion;NI, Neural invasion; CI, Confidence interval; OR, Odds ratio; ROC, Receiver operating characteristic (ROC); PPV, Positive predictive value; NPV, Negative predictive value; DMR, Differential methylation region.
