# Supplementary material for: A novel DNA methylation marker to identify lymph node metastasis of colorectal cancer
Source: Front Oncol. 2022 Oct 14;12:1000823. doi: 10.3389/fonc.2022.1000823 (PMC9614158; doi:10.3389/fonc.2022.1000823)
Supplement: Supplementary file 1 [file DataSheet_1.docx]

**Supplementary Materials**

**A**


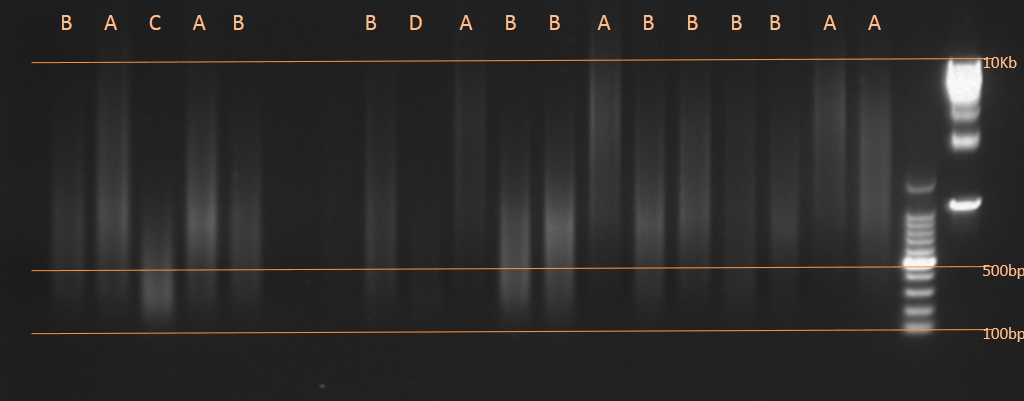


**B**


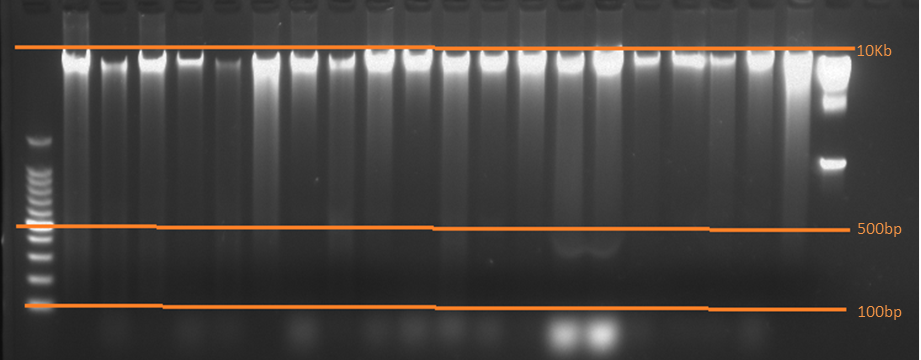


**Figure S1.** **DNA samples were controlled by agarose gel electrophoresis. A**. Level A:Qualified DNA samples demand the extracted DNA concentration was >10ng/ul, and the main band of agarose gel was evenly distributed between 500bp-10KB; Level B: Partial degradation of DNA: Although the DNA concentration was more than 10ng/ul and most of the main bands of agarose gel were distributed over 500bp, a few main bands were still distributed below 500bp. Level C: Unqualified DNA samples: Although THE DNA concentration was more than 10ng/ul, the main bands of agarose gel are distributed around 500bp, and more than half of the main bands are below 500bp; **B.**The main bands of all DNA samples were more than 500bp, and most were distributed around 10Kb.

**A B**


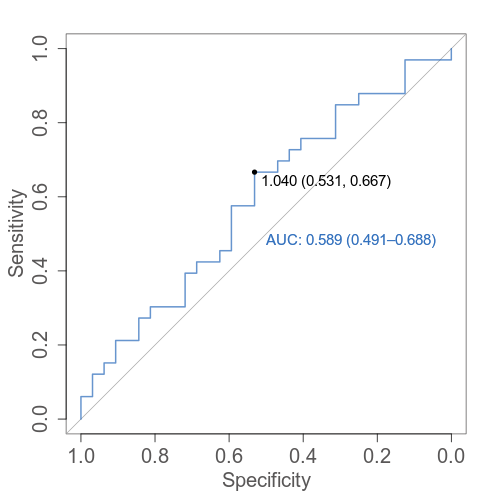

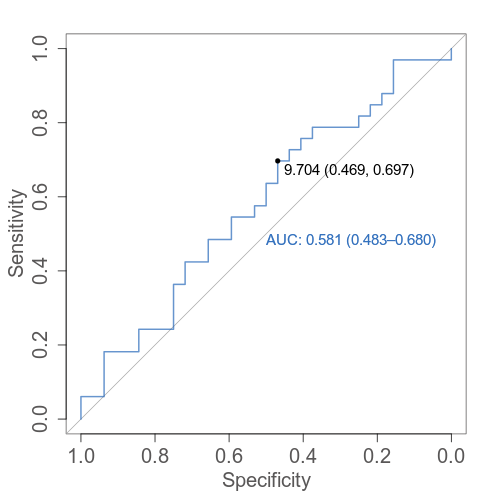


*ACHE RPS15*

**C D**


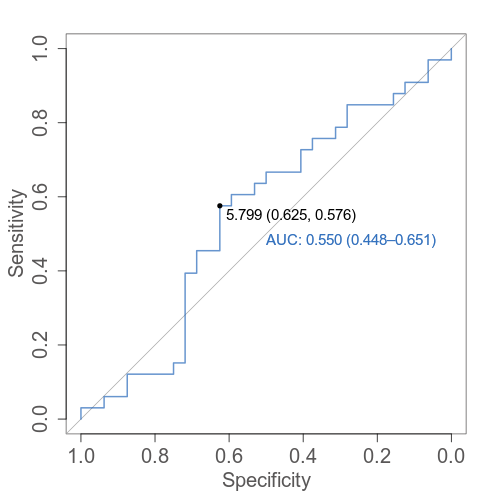

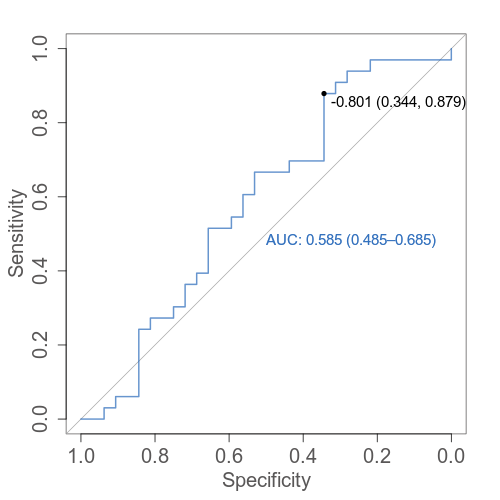


*APC2 BAHCC1*

**E** **F**


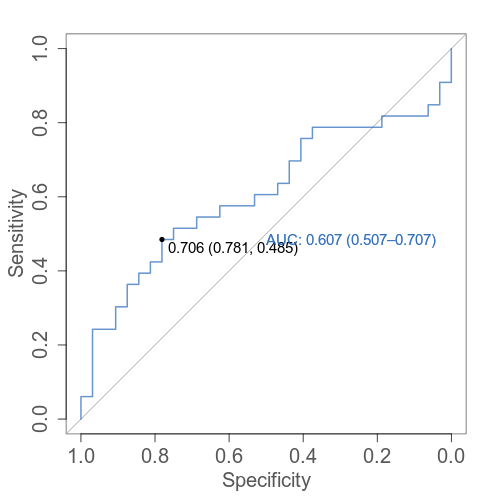

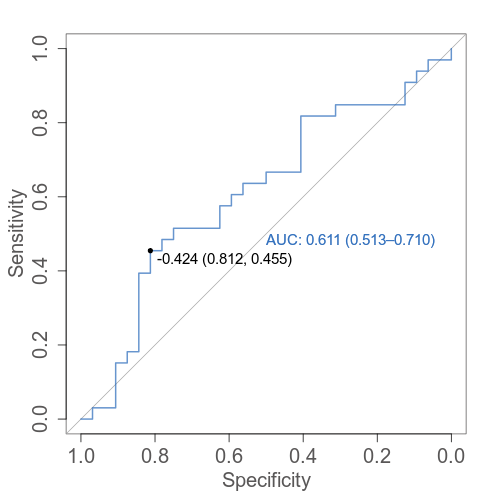


*LEFTY1 RTN4RL2*

**G**  **H**


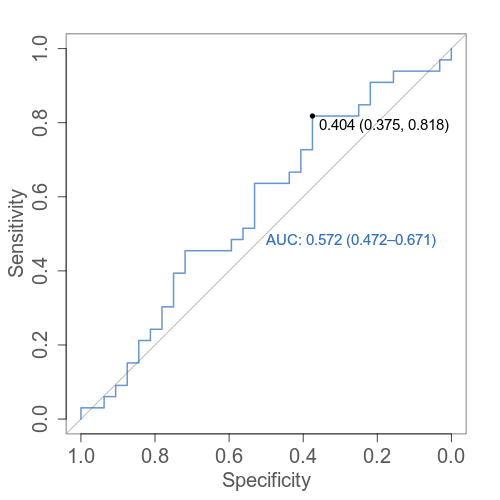

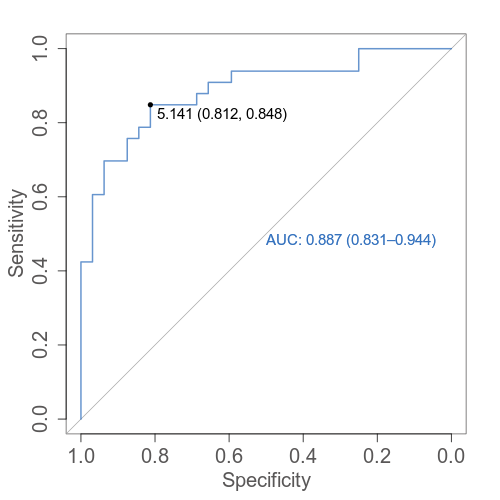


*KCNQ1 LBX2*

**I** **J**


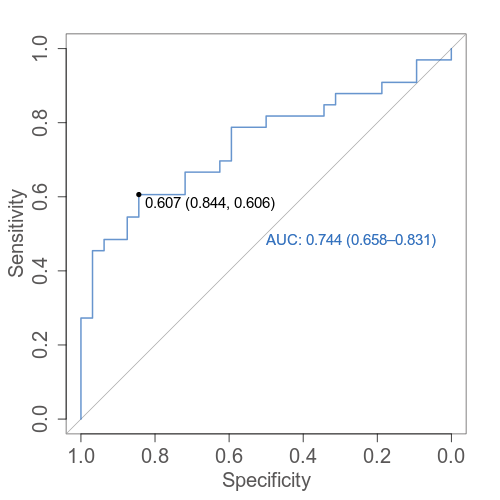

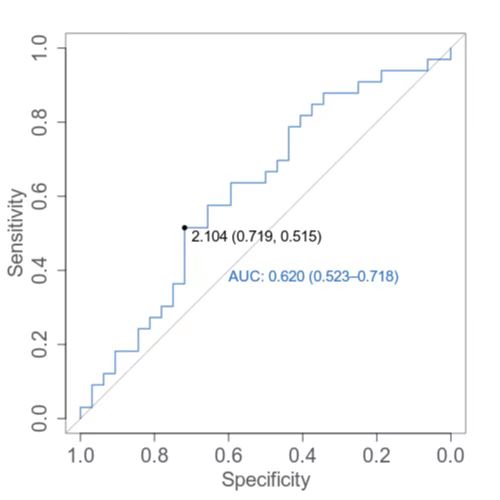


*STMN3 SS18L1*

**K** **L**


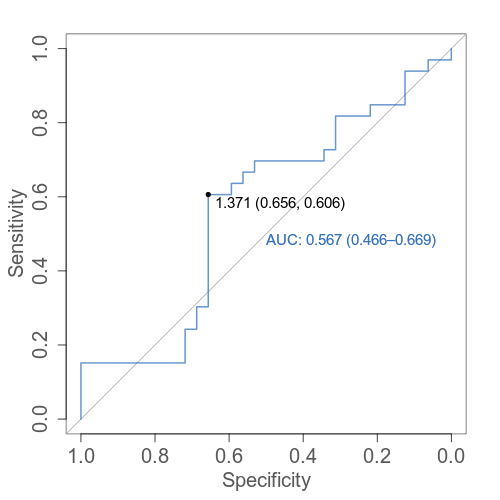

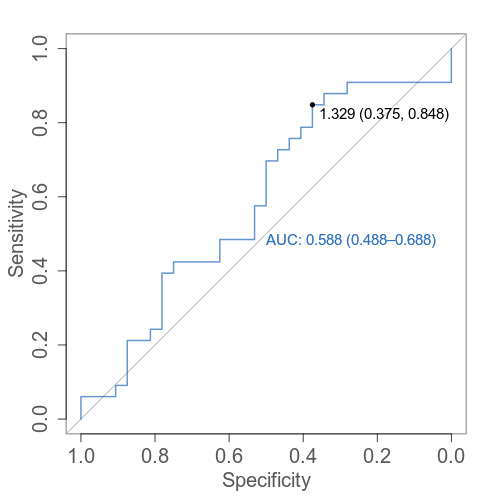


*CYTH2 LINC01072*

**Figure S2.** **ROC curves of 12 methylation markers(*ACHE、RPS15、APC2、BAHCC1、LEFTY1、RTN4RL2、KCNQ1、LBX2、STMN3、SS18L1、CYTH2、LINC01072*)**


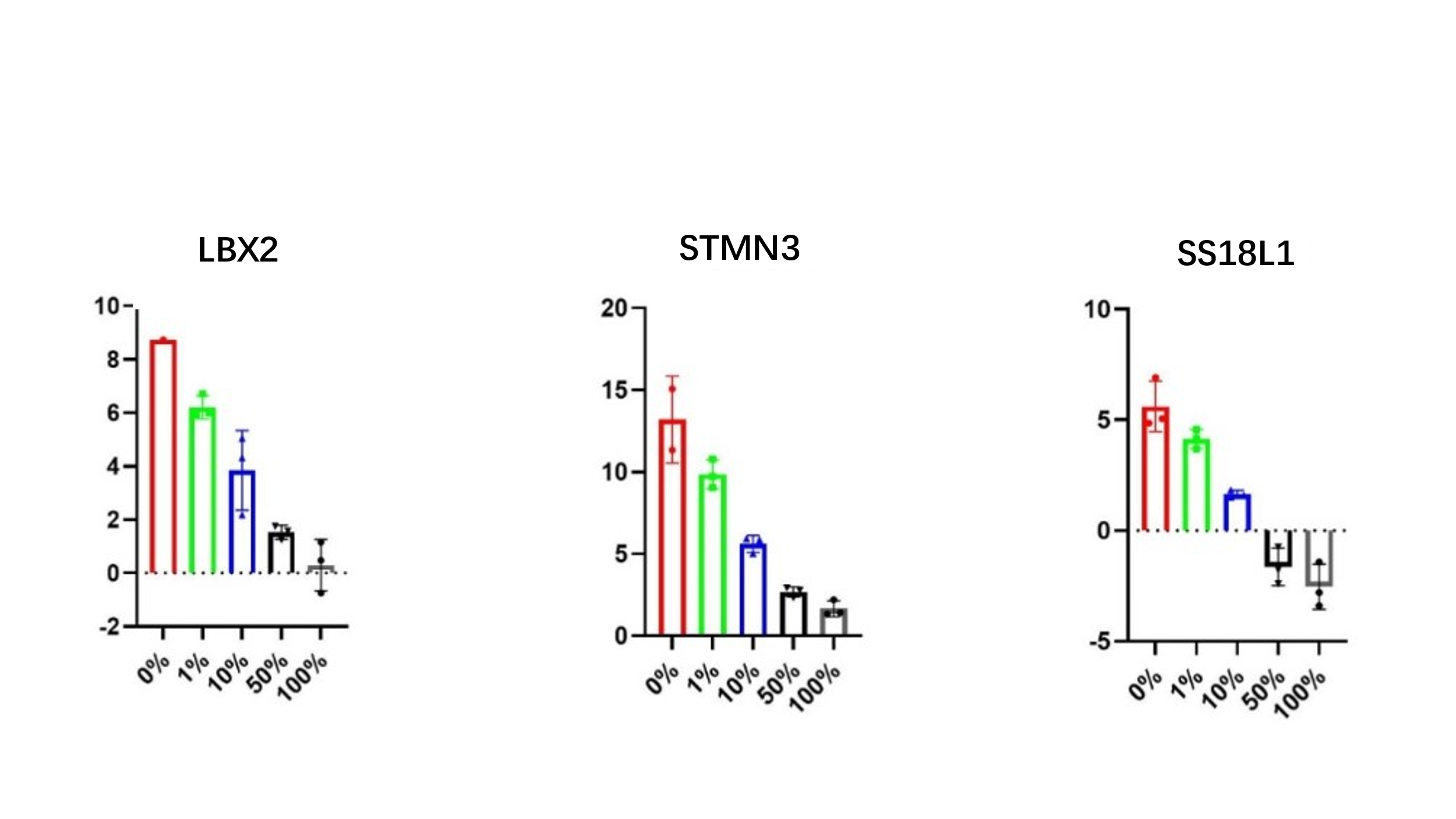


### Figure S3. Primer/ probes of *LBX2, STMN3* and *SS18L1* in different gradients concentrations could distinguish methylation levels. Methylight qPCR was used to verify the methylation degree of the three primers in five different methylation gradients (0%, 1%, 10%, 50%, 100%) of genomic DNA standard samples. The degree of methylation was inversely proportional to the respective PCR detla CT, and the concentration of methylated standard samples in five gradients was detla CT values showed a gradient decline, which indicatd that qPCR method could accurately reflect the methylation level of candidate markers.

|  |  |  |  |  |  |  |  |
| --- | --- | --- | --- | --- | --- | --- | --- |

**A** **B**

**C D**

**E F**

**G H**

**Figure S4.** **Relationship between clinicopathologic feature and LNM. A.** Relationship between gender and LNM.**B.** Relationship between age and LNM. **C.** Relationship between t-staging and LNM. **D.** Relationship between tumor size and LNM. **E.** Relationship between ulceration and LNM.**F.** Relationship between Lymp-vessel invasion and LNM.**G.** Relationship between vascular invasion and LNM. **H.** Relationship between Nerve invasion and LNM.

**Table S1. The information of EPIC database construction samples**

| **Library numbers** | **Sex** | | **Age** | | **Tunor size**  **(cm)** | **Ulceration** | **Lymph-vessel invasion** | **Blood vessel invasion** | **Nerve invasion** | **T-stage** | **LNM(-/+)** |
| --- | --- | --- | --- | --- | --- | --- | --- | --- | --- | --- | --- |
| EPIC-CRC-001-FR-IRM | | Male | | 78 | 2.0*1.2*0.4 | （+） | （+） | （-） | （-） | 3 | LNM+ |
| EPIC-CRC-014-FR-IRM | | Female | | 67 | 3.2*2.8*0.6 | （+） | （-） | （-） | （+） | 3 | LNM+ |
| EPIC-CRC-015-FR-IRM | | Female | | 61 | 3.0*2.3*0.4 | （+） | （-） | （-） | （+） | 4a | LNM+ |
| EPIC-CRC-019-FR-IRM | | Male | | 61 | 6.0*3.0*1.5 | （-） | （-） | （-） | （+） | 3 | LNM+ |
| EPIC-CRC-024-FR-IRM | | Female | | 69 | 6.0*5.5*3.0 | （+） | （-） | （-） | （-） | 3 | LNM+ |
| EPIC-CRC-030-FR-IRM | | Male | | 56 | 4.5*4.0*0.7 | （+） | （+） | （+） | （+） | 3 | LNM+ |
| EPIC-CRC-031-FR-IRM | | Male | | 70 | 2.0*2.0*1.5 | （-） | （-） | （-） | （-） | 3 | LNM+ |
| EPIC-CRC-041-FR-IRM | | Female | | 64 | 6.5*6.0*5.0 | （-） | （+） | （+） | （+） | 4a | LNM+ |
| EPIC-CRC-051-FR-IRM | | Female | | 55 | 4.0*3.0*2.0 | （-） | （-） | （-） | （-） | 3 | LNM+ |
| EPIC-CRC-053-FR-IRM | | Male | | 80 | 10.0*3.5*2.5 | （-） | （-） | （-） | （-） | 4 | LNM+ |
| EPIC-CRC-054-FR-IRM | | Female | | 65 | 3.5*2.0*1.0 | （+） | （-） | （-） | （+） | 3 | LNM+ |
| EPIC-CRC-058-FR-IRM | | Male | | 63 | 7.0*3.0*1.5 | （+） | （+） | （+） | （+） | 3 | LNM+ |
| EPIC-CRC-061-FR-IRM | | Male | | 55 | 4.5*2.5*1.2 | （-） | （+） | （+） | （+） | 3 | LNM+ |
| EPIC-CRC-063-FR-IRM | | Female | | 46 | 3.5*5.0*0.5 | （-） | （-） | （-） | （-） | 3 | LNM+ |
| EPIC-CRC-069-FR-IRM | | Male | | 64 | 3.0*2.5*1.0 | （+） | （-） | （-） | （+） | 3 | LNM+ |
| EPIC-CRC-082-FR-IRM | | Male | | 77 | 3.0*2.0*1.2 | （+） | （-） | （-） | （-） | 4a | LNM+ |
| EPIC-CRC-086-FR-IRM | | Female | | 64 | 3.0*2.0*1.0 | （+） | （-） | （-） | （-） | 3 | LNM+ |
| EPIC-CRC-087-FR-IRM | | Male | | 57 | 4.0*3.0*2.0 | （-） | （-） | （-） | （） | 4b | LNM+ |
| EPIC-CRC-100-FR-IRM | | Male | | 68 | 7.0*6.5*0.5 | （-） | （-） | （-） | （+） | 3 | LNM+ |
| EPIC-CRC-018-FR-IRM | | Female | | 67 | 3.5*2.5*0.7 | （-） | （-） | （-） | （-） | 3 | LNM- |
| EPIC-CRC-023-FR-IRM | | Male | | 57 | 2.8*2.8*0.8 | （-） | （-） | （-） | （-） | 3 | LNM- |
| EPIC-CRC-012-FR-IRM | | Male | | 51 | 4.0*3.0*2.5 | （+） | （-） | （-） | （+） | 4a | LNM- |
| EPIC-CRC-052-FR-IRM | | Female | | 66 | 3.0*2.0*0.5 | （+） | （-） | （-） | （-） | 3 | LNM- |
| EPIC-CRC-066-FR-IRM | | Female | | 78 | 4.0*2.5*2.0 | （+） | （-） | （-） | （+） | 3 | LNM- |
| EPIC-CRC-067-FR-IRM | | Female | | 56 | 4.0*2.0*1.0 | （-） | （-） | （-） | （+） | 4a | LNM- |
| EPIC-CRC-071-FR-IRM | | Male | | 76 | 5.0*4.3*0.5 | （+） | （+） | （+） | （+） | 3 | LNM- |
| EPIC-CRC-078-FR-IRM | | Male | | 61 | 4.5*3.5*1.5 | （-） | （-） | （-） | （+） | 3 | LNM- |
| EPIC-CRC-081-FR-IRM | | Male | | 85 | 5.0*4.0*2.5 | （-） | （-） | （-） | （+） | 3 | LNM- |
| EPIC-CRC-098-FR-IRM | | Male | | 65 | 5.0*4.0*2.0 | （+） | （-） | （-） | （+） | 3 | LNM- |
| EPIC-CRC-099-FR-IRM | | Female | | 66 | 3.0*3.0*1.5 | （+） | （-） | （-） | （-） | 3 | LNM- |

|  |
| --- |
| **Table S2.** **Quality inspection results after library sequencing** |


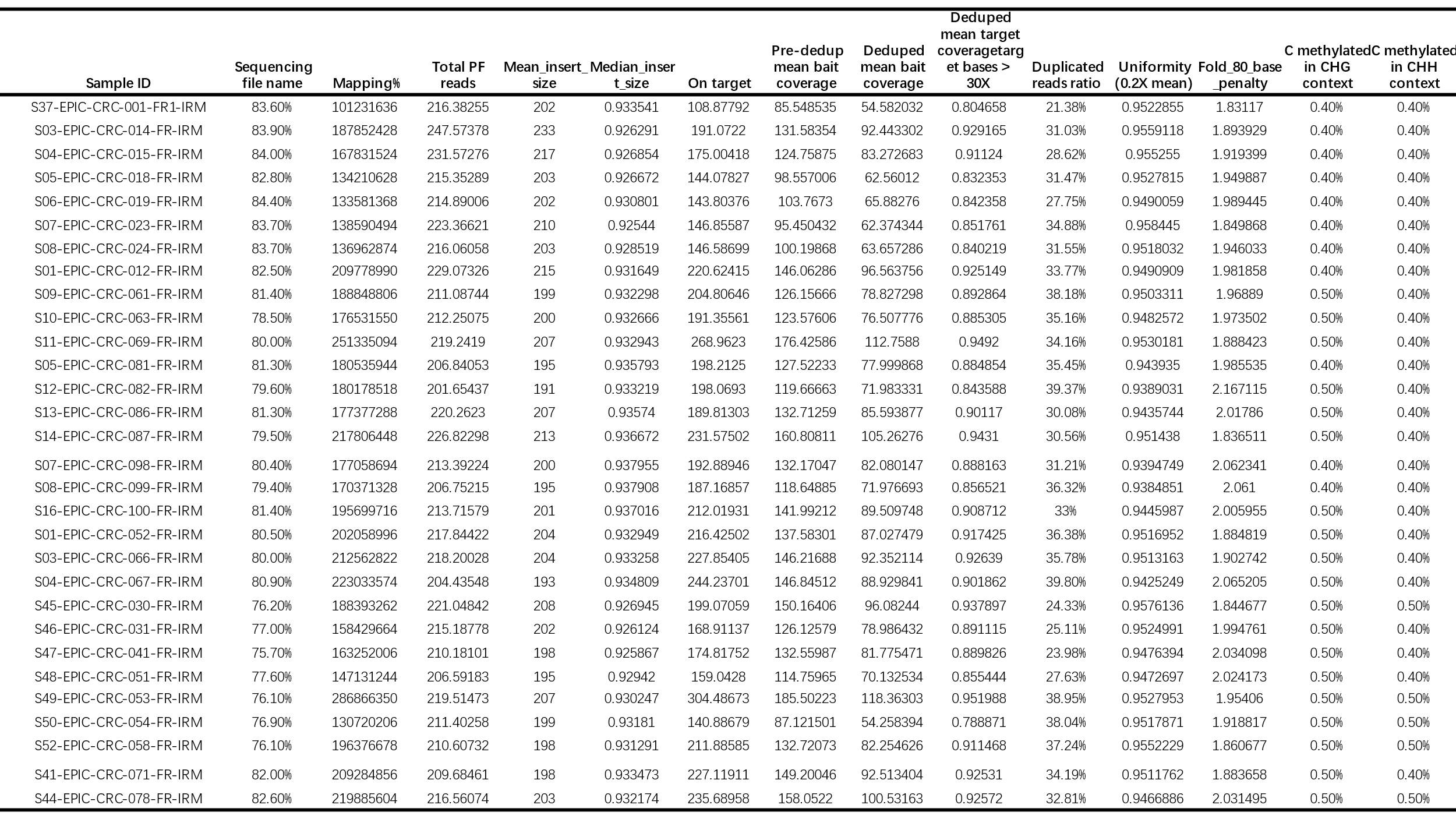


|  |  |  |  |  |  |  |  |  |
| --- | --- | --- | --- | --- | --- | --- | --- | --- |
